# VEGF receptor antagonist Cyclo-VEGI reduces inflammatory reactivity and vascular leakiness and is neuroprotective against acute excitotoxic striatal insult

**DOI:** 10.1186/1742-2094-5-18

**Published:** 2008-05-20

**Authors:** Jae K Ryu, James G McLarnon

**Affiliations:** 1 Department of Anesthesiology, Pharmacology and Therapeutics, Faculty of Medicine, University of British Columbia, Vancouver, BC V6T 1Z3, Canada

## Abstract

**Background:**

Excitotoxic brain insult is associated with extensive neuronal damage but could also cause inflammatory reactivity and vascular remodeling. The effects of the vascular endothelial growth factor (VEGF) inhibitor, Cyclo-VEGI on expression of VEGF, microgliosis and astrogliosis, blood-brain barrier (BBB) integrity and neuronal viability have been studied following intra-striatal injection of the excitotoxin, quinolinic acid (QUIN). The purpose of this study was to examine VEGF-dependent inflammatory responses in excitotoxin-injected brain and their dependence on pharmacological antagonism of VEGF receptors.

**Methods:**

Single and double immunofluorescence staining of cellular (microglia, astrocyte, neuron) responses and dye and protein infiltration of blood-brain barrier have been applied in the absence, and presence, of pharmacological modulation using a VEGF receptor antagonist, Cyclo-VEGI. Dunn-Bonferroni statistical analysis was used to measure for significance between animal groups.

**Results:**

Detailed analysis, at a single time point of 1 d post-QUIN injection, showed excitotoxin-injected striatum to exhibit marked increases in microgliosis (ED1 marker), astrogliosis (GFAP marker) and VEGF expression, compared with PBS injection. Single and double immunostaining demonstrated significant effects of Cyclo-VEGI treatment of QUIN-injected striatum to inhibit microgliosis (by 38%), ED1/VEGF (by 42%) and VEGF striatal immunoreactivity (by 43%); astrogliosis and GFAP/VEGF were not significantly altered with Cyclo-VEGI treatment. Leakiness of BBB was indicated by infiltration of Evans blue dye and plasma protein fibrinogen into QUIN-injected striatum with barrier permeability restored by 62% (Evans blue permeability) and 49% (fibrinogen permeability) with Cyclo-VEGI application. QUIN-induced toxicity was demonstrated with loss of striatal neurons (NeuN marker) and increased neuronal damage (Fluoro-Jade marker) with significant neuroprotection conferred by Cyclo-VEGI treatment (33% increase in NeuN and 38% decrease in Fluoro-Jade).

**Conclusion:**

An antagonist for VEGF receptor-mediated signaling, Cyclo-VEGI, has shown efficacy in a broad spectrum of activity against striatal excitotoxic insult including inhibition of microgliosis, reduction in leakiness of BBB and parenchymal infiltration of plasma fibrinogen and in conferring significant protection for striatal neurons. Antagonism of VEGF-mediated activity, possibly targeting VEGF receptors on reactive microglia, is suggested as a neuroprotective mechanism against inflammatory reactivity and a novel strategy to attenuate acute excitotoxic damage.

## Background

Excitotoxicity has been implicated as a contributing factor in the pathogenesis of neurological disorders [1,2]. Although excitotoxic insult directly induces neuronal damage through activation of glutamate subtype receptors, results from several studies have suggested excitotoxin-induced inflammatory processes could also indirectly contribute to loss of neuron viability [3-7]. A rapid enhancement of a spectrum of proinflammatory mediators including cytokines, enzymes and free radicals have been reported following excitotoxic brain insult [8-11]. Resident glial cells, microglia and astrocytes, are a likely source of the inflammatory factors [6,10,12,13].

Glial-derived factors can also cause rapid changes in vascular processes and altered vasculature is a prominent feature of inflammatory responses in pathological conditions including excitotoxicity [14]. Vascular endothelial growth factor (VEGF) is a potent glial-derived stimulator of vascular remodeling in various tissues with both the VEGFR-1 (Flt-1) and VEGFR-2 (KDR/Flk-1)-type receptors expressed by endothelial cells. Evidence suggests VEGFR-2 have critical functions in mediating angiogenic [15] and neurogenic [16] activity. In contrast, the VEGFR-1 subtype is predominantly expressed by microglia and astrocytes and contributes to cellular chemotactic responses [17,18]. VEGF-dependent signaling in brain has been associated with both neuroprotection and neurotoxicity [19-21] which could reflect differential effects of the factor in binding to VEGF receptors on neurons, blood vessels or glial cells.

The primary questions addressed in the present study were the roles of microglial VEGF receptor and microglial immunoreactivity in linking striatal excitotoxic insult with vascular perturbations and neuronal damage. Initial studies demonstrated a considerable extent of excitotoxic lesion occurred at 1 d post-striatal injection of quinolinic acid (QUIN) and detailed analysis was carried out at this time point. Effects of the VEGF receptor antagonist Cyclo-VEGI were determined on VEGF expression, gliosis, permeability of Evans blue dye and plasma protein fibrinogen through blood-brain barrier (BBB) and as a pharmacological modulator of neuronal viability. The overall results suggest microglial-derived VEGF as a critical factor in mediating inflammatory reactivity and linking excitotoxic insult with vascular abnormalities and neuronal degeneration.

## Methods

### Animals

Adult male Sprague-Dawley rats (Charles River Laboratories, St. Constant, Quebec, Canada) weighting 250–300 g were used in this study. The rats were housed in a temperature and humidity controlled environment under a 12-hr light-dark cycle with food and water available *ad libitum*. All experimental procedures were approved by the University of British Columbia Animal Care Ethics Committee, adhering to guidelines of the Canadian Council on Animal Care.

### Administration of quinolinic acid (QUIN) and Cyclo-VEGI

Animals were anesthetized with intraperitoneal (i.p.) injection of a mixture of ketamine hydrochloride (72 mg/kg; Bimeda-MTC, Cambridge, Ontario, Canada) and xylazine hydrochloride (9 mg/kg; Bayer Inc., Etobicoke, Ontario, Canada) and then placed in a stereotaxic apparatus (David Kopf Instruments, Tujunga, CA). Intrastriatal injection of quinolinic acid (QUIN) or PBS was performed as previously described [6]. In brief, animals received unilateral injection of 1 μl QUIN (60 nmol; Sigma, St. Louis, MO) over 4 min using a 10 μl Hamilton syringe fitted with a 26-gauge needle at the following coordinate: AP: +1.0 mm, ML: -3.0 mm, DV: -5.0 mm, from bregma [22]. The injection syringe was left in place for an additional 4 min to allow the QUIN to diffuse from the needle tip. After removing the needle, the skin was sutured and the animals were allowed to recover and then returned to their cages.

### Cyclo-VEGI

The compound Cyclo-VEGI (Calbiochem, La Jolla, CA) is a competitive antagonist acting at both the VEGF-2 (KDR) and VEGF-1 (Flt-1) receptors [23]. The compound was first dissolved in endotoxin-free water (Sigma) and further diluted in PBS. Cyclo-VEGI (100 μM), in a volume of 4 μl, was administered into right lateral ventricle (AP: -0.3 mm, ML: -1.2 mm, DV: -4.0 mm from bregma) 30 min prior to intrastriatal QUIN injection under the anesthesia. QUIN-injected rats received lateral ventricular injection of PBS vehicle. The dose and administration protocol of Cyclo-VEGI followed published work [24,25].

### Tissue preparation

Animals were deeply anesthetized with a mixture of ketamine and xylazine and then perfused transcardially with heparinized cold saline followed by 4% paraformaldehyde in 0.1 M phosphate buffer (0.1 M PB, pH 7.4). The brains were removed from the skull and postfixed in the same fixative solution and then placed in 30% sucrose for cryoprotection. The brains were then frozen in powdered dry ice and stored at -70°C. Coronal brain sections (40 μm) were cut throughout the striatum on a cryostat and the sections were stored in cryoprotectant solution.

### Immunohistochemistry

Free-floating sections were processed for the immunohistochemistry as described previously [6,7]. Briefly, endogenous peroxidase was quenched with 3% hydrogen peroxide in 0.1 M PBS and sections were incubated in blocking solution containing 10% normal goat serum (NGS) and 0.2% Triton X-100 in 0.1 M PBS for 30 min. The sections were then incubated at 4°C for 24 hr with the following primary antibody against neuronal nuclei (NeuN, 1:1000; Chemicon, Temecula, CA) or vascular endothelial growth factor (VEGF, 1:200; Santa Cruz Biotechnology, Santa Cruz, CA). Sections were incubated at room temperature (RT) for 1 hr with biotinylated anti-mouse or anti-rabbit IgG (1:1000; Vector, Burlingame, CA), followed by an avidin-biotin-peroxidase complex (ABC, 1:1000; Vector) for 1 hr. Reaction products were visualized with 3,3'-diaminobenzidine (DAB, Sigma) and hydrogen peroxide. Sections were washed in 0.1 M PB, placed on Superfrost/Plus microscope slides (Fisher Scientific; Pittsburgh, PA), dehydrated, and mounted in Mountant (Fluka, Toronto, Canada). For immunofluorescence staining, sections were incubated with antibodies against the following: ED1 (1:1000; Serotec, oxford, UK; a marker for activated microglia/macrophages), glial fibrillary acidic protein (GFAP, 1:1000; Sigma; a marker for astrocytes), rat endothelial cell antigen-1 (RECA-1, 1:500; Serotec; a marker for endothelial cells), fibrinogen (1:2000; DAKO, Carpinteria, CA) or NeuN (1:1000; Chemicon) followed by Alexa Fluor-conjugated anti-mouse or anti-rabbit IgG (1:500; Molecular Probes, Eugene, OR) at RT for 2 hr in the dark. Omission of the primary antibody was used as a negative control.

### Double immunofluorescence staining

Double immunofluorescence staining was performed as described previously [10]. In brief, sections were blocked for 30 min with 10% NGS and incubated overnight at 4°C with the following primary antibodies: anti-VEGF (1:200; Santa Cruz Biotechnology) in combination with anti-ED1 (1:500; Serotec) or anti-GFAP (1:1000; Sigma). In another experiment, sections were incubated with a mixture of anti-VEGFR-1 (1:500; Santa Cruz Biotechnology; a marker for VEGF receptor-1) and anti-ED1 (1:500; Serotec). Sections were then incubated in a mixture of Alexa Fluor-conjugated 488 anti-rabbit IgG (1:500; Molecular Probes) and Alexa Fluor-conjugated 594 anti-mouse IgG (1:500; Molecular Probes) at RT for 2 hr in the dark.

### Image analysis

The five matched striatal sections (spaced 200 μm apart) from each animal were used in the analysis. These sections were representative of QUIN striatal injury as determined by ED1 (microglia), GFAP (astrocytes), and NeuN (neurons) immunohistochemistry [6]. All images were examined under a Zeiss Axioplan 2 fluorescent microscope (Zeiss, Jena, Germany) using a DVC camera (Diagnostic Instruments, Sterling Heights, MI, USA) and analyses were performed with Northern Eclipse software (Empix Imaging, Mississauga, ON, Canada). In each section, four non-overlapped fields were randomly selected at a final magnification of 400×. All quantitative analyses were carried out in a blinded manner. The density of ED1, GFAP, and NeuN immunoreactive cells in the striatum was evaluated by counting of immunoreactive cells and expressed as the number of cells/mm^2^. Fluoro-Jade B stained sections were visualized under a fluorescence microscope using a fluoresceine (FITC) filter and Fluoro-Jade B fluorescent cells were quantified and expressed as cells/mm^2^. Glial expression of VEGF was measured by counting the number (cells/mm^2^) of double labeled ED1 and GFAP labeled for VEGF. For quantification of VEGF or fibrinogen immunohistochemical staining, percentage of total area exhibiting positive immunoreactivity was measured as described previously [26].

### Measurement of blood-brain barrier permeability

We used two approaches to examine integrity of blood-brain barrier (BBB). One method measures extravasation of the plasma protein fibrinogen into rat striatum. Standard immunohistochemical staining used antibody to fibrinogen (see above) to quantify extents of area density of striatal fibrinogen with the different animal treatments. A second method determined infiltration of Evans blue dye through a leaky BBB and followed published procedures [26,27]. Briefly, Evans blue dye (2%, Sigma) was administered by i.p. injection and animals sacrificed at 1 hr after the injection of the solution. Evans blue dye leakage into brain tissue was visualized on five striatal brain sections (spaced 200 μm apart) under a fluorescent microscope to determine fluorescent intensity [26].

### Analysis for neuronal viability

Striatal neuronal damage was assessed by both immunohistochemical staining for neurons (use of NeuN as a marker, see above) and Fluoro-Jade B staining. In the former case, NeuN staining measured numbers of striatal neurons with the different animal treatments. Fluoro-Jade B staining, a marker for degenerating neurons, was used following published protocols [6,28]. In brief, striatal sections were mounted on coated glass slides and incubated in 0.06% potassium permanganate. The slides were then incubated in 0.001% Fluoro-Jade B (Histo-Chem, Jefferson, AR) for 20 min and washed in distilled water.

### Statistical analysis

All data are expressed as means ± SEM. Statistical significance of differences for group comparisons was assessed by analysis of variance followed by the Dunn-Bonferroni test. Significance was set at *p *< 0.05.

## Results

### Time dependent changes of gliosis and effects of the VEGF antagonist Cyclo-VEGI in QUIN-injected rat striatum

**Figure 1:**
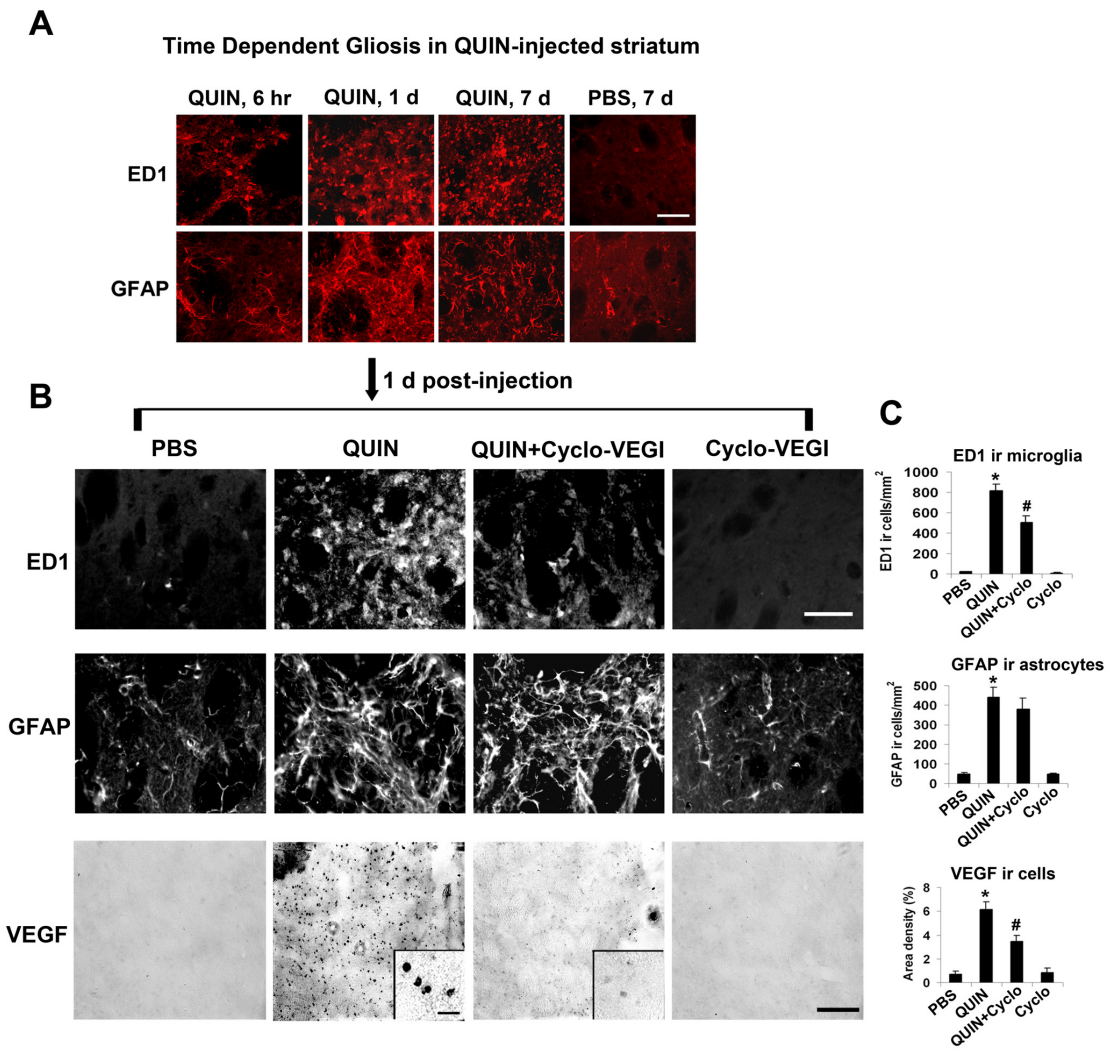
**Time-dependent gliosis and effects of Cyclo-VEGI on microgliosis, astrogliosis and VEGF expression at 1 d post QUIN-intrastriatal injection**. (A) Representative staining for microglia (ED1 marker) and astrocytes (GFAP marker) at 6 hr, 1 d and 7 d QUIN-injection; typical marker immunoreactivities (ir) for control PBS (7 d injection) are also shown. Scale bar represents 150 μm. (B) Photomicrographs of immunofluorescence staining for different injection protocols (1 d intra-striatal injection). Typical patterns of staining are shown for ED1 ir microglia (upper panels), GFAP ir astrocytes (middle panels), and VEGF expression (lower panels). Scale bar represents 60 μm for ED1 panel and 300 μm for VEGF panel. The insets in VEGF panels show cell-associated VEGF; scale bar = 25 μm. (C) Bar graphs for ED1 (upper), GFAP (middle) and VEGF (lower) ir. Data are mean ± SEM (n = 5 animals/group). *p < 0.05 compared with PBS-injected group. ^#^*p *< 0.05 compared with QUIN-injected group.

Initial experiments were designed to assess time-dependent changes in microgliosis and astrogliosis induced by striatal injection of QUIN. Representative staining shows microglial and astrocytic immunoreactivities (ir, markers ED1 and GFAP, respectively) for QUIN injections of 6 hr, 1 d and 7 d and also 7 d PBS control (Fig. 1A). Both ED1 and GFAP ir were increased with QUIN stimulation from 6 hr to 1 d, however, both markers demonstrated no additional increases for the longest duration of QUIN injection at 7 d. At this time, PBS injected striatum showed low levels of microgliosis and astrogliosis. Although data were not quantified, the results suggested 1 d QUIN injection as a time point for detailed analysis of gliosis and putative VEGF-dependent activity in excitotoxin-injected rat brain.

We next examined effects of the VEGF antagonist, Cyclo-VEGI (intraventricle administration at 100 μM) on extents of gliosis and VEGF ir with intrastriatal QUIN injection (1 d). Marked increases in both ED1 and GFAP ir were evident following QUIN compared with PBS control (upper and middle left panels, Fig. 1B). Representative immunostaining showed microgliosis, but not astrogliosis, was attenuated with Cyclo-VEGI treatment in QUIN-injected striatum with the VEGF antagonist alone showing gliosis ir similar to control (upper and middle right panels, Fig. 1B). Quantification of data is presented in Fig. 1C (n = 5 animals/group), with Cyclo-VEGI treatment exhibiting a significant reduction in ED1 ir (by 38%) when injected with QUIN compared with QUIN stimulation alone (Fig. 1C, upper bar graph). Although Cyclo-VEGI decreased GFAP ir by 13% in QUIN-injected striatum, the change was not significant (Fig. 1C, middle bar graph). Application of Cyclo-VEGI alone was associated with similar patterns of gliosis as measured with PBS control striatal injection.

Levels of striatal VEGF were considerably elevated in QUIN-injected rat striatum compared with PBS injection (Fig. 1B, lower left panels). Cyclo-VEGI was effective in diminishing VEGF ir when applied in QUIN-injected striatum and showed no induced VEGF ir when applied alone (Fig. 1B, lower right panels). Higher magnification insets shows typical VEGF staining in association with cells in QUIN and QUIN + Cyclo-VEGI injected striatum. VEGF ir cells exhibited a microglial-like cellular morphology particularly evident with QUIN injection; this point was investigated using double staining (see below). Overall (n = 5 animals/group), VEGF ir was increased 8-fold with QUIN, relative to PBS, injection (Fig. 1C, lower bar graph). Cyclo-VEGI exhibited a significant reduction in VEGF ir (by 43%) in QUIN-injected striatum. Levels of striatal VEGF were similar with injection of PBS or Cyclo-VEGI applied alone.

### Effects of Cyclo-VEGI on glial-cell associated VEGF

**Figure 2:**
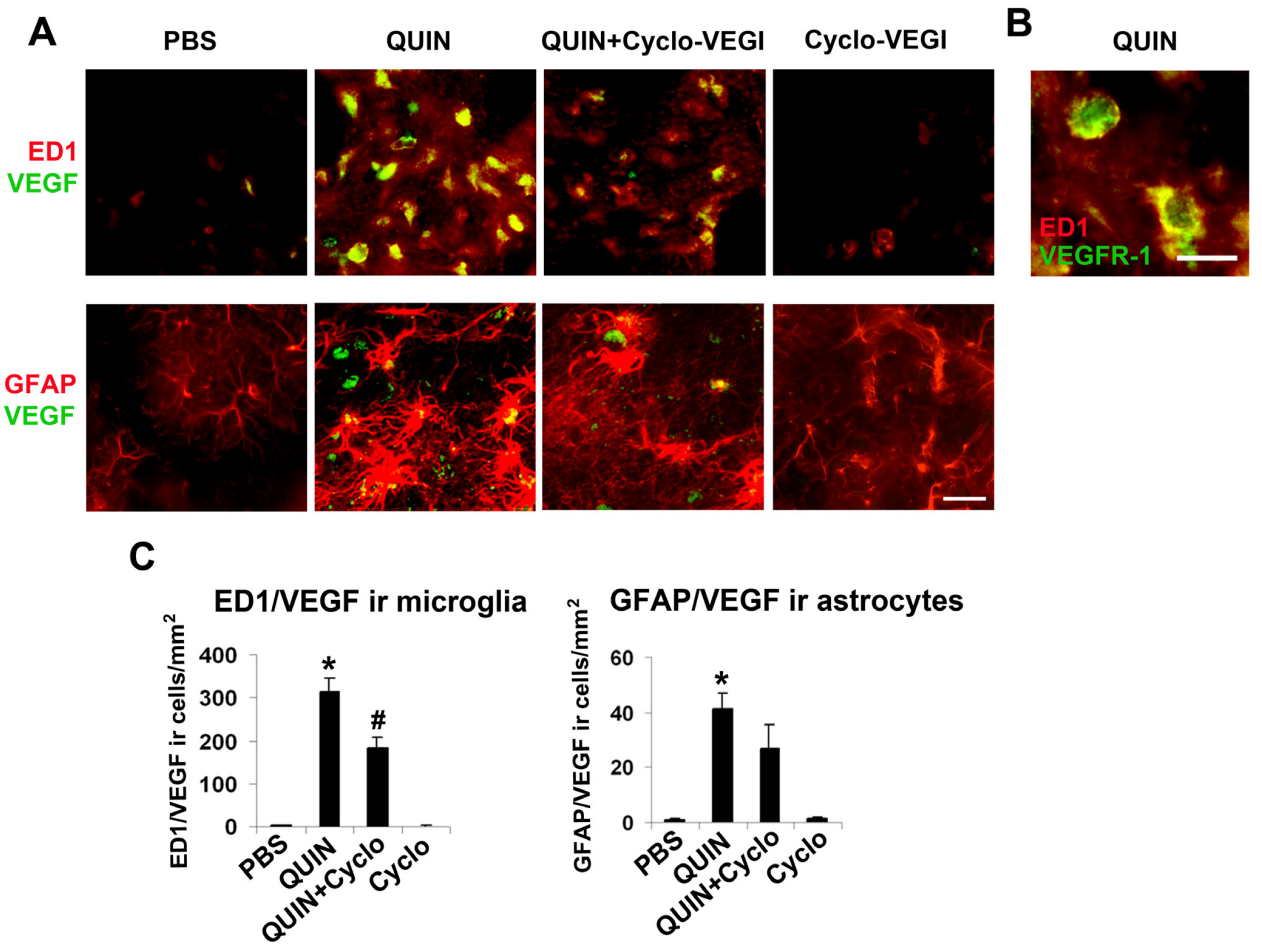
**Effects of Cyclo-VEGI on VEGF association with glial cells**. (A) Double staining for ED1/VEGF (upper panels) and GFAP/VEGF (lower panels) after 1 d injections of PBS (left panel), QUIN (left middle panel), QUIN with Cyclo-VEGI (right middle panel) and Cyclo-VEGI (right panel). Scale bar = 50 μm. (B) Representative examples of merged staining for VEGFR-1 with ED1 (+)ve microglia. Scale bar = 15 μm. (C) Quantitative analysis for the area density of ED1/VEGF (left bar graph) and GFAP/VEGF (right bar graph). Data are mean ± SEM (n = 5 animals/group). *P < 0.05 compared with PBS-injected group. ^#^*P *< 0.05 compared with QUIN-injected group.

As noted, QUIN-injected rat striatum demonstrated a pattern of VEGF association with glia with cells exhibiting a roundish appearance indicative of activated microglia (insets, lower panel, Fig. 1B). In order to address this point, double immunohistochemical staining was used to examine VEGF localization with ED1 (+)ve microglia or GFAP (+)ve astrocytes. Representative double staining shows ED1/VEGF ir with the different treatments in Fig. 2A (upper row). Considerable extents of VEGF ir were associated with microglia in QUIN-injected striatum. Double staining of ED1/VEGF was diminished with Cyclo-VEGI treatment of QUIN-injected animals with the VEGF receptor antagonist alone showing a similar double staining pattern as for PBS control. As shown in Fig. 2B, ED-1 (+)ve microglia stain positively for VEGF receptor-1 (VEGFR-1) in QUIN-injected brain. Limited analysis of brain tissue estimated in excess of 90% of microglia expressed the VEGFR-1 marker. Interestingly, microglia demonstrated a highly reactive morphology of rounded cell bodies and retracted processes with excitotoxic insult. No VEGFR-1 immunoreactivity was observed with GFAP (+)ve astrocytes (data not shown). Representative GFAP/VEGF staining patterns for the different animal groups are shown in Fig. 2A (lower row). QUIN-injected brain showed evidence for VEGF localization with astrocytes which was reduced with Cyclo-VEGI treatment of excitotoxin-injected brain. Cyclo-VEGI alone demonstrated a pattern of GFAP/VEGF similar to PBS control.

Overall (n = 5 animals/group), Cyclo-VEGI significantly inhibited ED1/VEGF ir by 42% (Fig. 2C, left bar graph). A comparison of single ED1 staining (Fig. 1C, upper bar graph) with double ED1/VEGF staining (Fig. 2C, left bar graph) suggests about 40% of total microglia were associated with VEGF in QUIN-injected striatum. Quantification of data (Fig. 2C, right bar graph) showed a 31% reduction in GFAP/VEGF ir with Cyclo-VEGI application to QUIN-stimulated striatum, however, this decrease did not reach significance (n = 5 animals/group). Cyclo-VEGI applied alone showed low levels of GFAP/VEGF ir. A comparison of GFAP single staining (Fig. 1C, middle bar graph) with GFAP/VEGF double staining (Fig. 2C, right bar graph) would suggest that less than 10% of astrocytes were associated with VEGF.

### Effects of Cyclo-VEGI on BBB permeability

**Figure 3:**
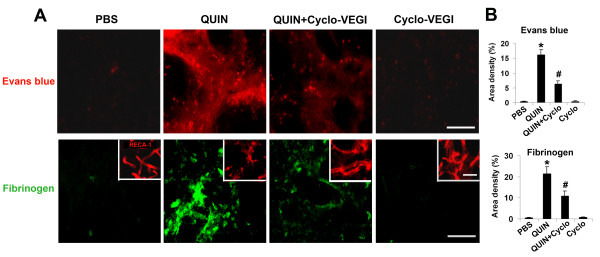
**Effects of Cyclo-VEGI on intactness of BBB**. (A) Photomicrographs of representative fluorescence images of Evans blue dye (upper panels) and fibrinogen (lower panels) with the different striatal injections (1 d post-injection): PBS (left panel), QUIN-injection (left middle panel), QUIN-injected rats with Cyclo-VEGI (right middle panel) and Cyclo-VEGI alone (right panel) treatment. Scale bar represents 500 μm. The insets in fibrinogen panels show typical RECA-1 ir; scale bars represent 150 μm for fibrinogen panel and 100 μm for RECA-1 panel. (B) Quantitative analysis of Evans blue dye (upper graph) and fibrinogen (lower graph) fluorescence intensity for the different procedures. Data are mean ± SEM (n = 5 animals/group). *P < 0.05 vs. PBS-injected group. ^#^*P *< 0.05 vs. QUIN-injected group.

We next designed experiments to test permeability of BBB, in the absence and presence of Cyclo-VEGI administration, in QUIN-injected striatum. Two different approaches were used; infiltration of Evans blue dye and extravasation of the plasma protein, fibrinogen. In the latter case, we also analyzed for fibrinogen ir in conjunction with endothelial cells using the endothelial specific cell marker RECA-1 (rat endothelial cell antigen). Typical Evans blue dye fluorescence for the different injections are presented in Fig. 3A (upper panels) and show considerable dye present in QUIN-injected brain but absent in PBS control. Dye accumulation in striatum was markedly attenuated with Cyclo-VEGI administration in QUIN-injected animals and was absent with Cyclo-VEGI treatment alone. Overall (n = 5 animals/group), Cyclo-VEGI was effective in reducing Evans blue fluorescence (by 62%) when applied in excitotoxin-injected animals (Fig. 3B). Low Evans blue fluorescence was measured in PBS control or with Cyclo-VEGI application alone.

Representative staining for fibrinogen are shown in Fig. 3A (lower panels). No fibrinogen ir was observed with PBS injection but high levels of striatal plasma protein were evident following QUIN injection. Cyclo-VEGI treatment was effective in attenuating fibrinogen ir in excitotoxin stimulated brain with no effect when applied alone. In total (n = 5 animals/group), Cyclo-VEGI demonstrated significant effects to attenuate levels of fibrinogen ir (by 49%) when administered with QUIN. Little or no fibrinogen ir accompanied Cyclo-VEGI administration alone.

Typical patterns of RECA-1 ir are presented in insets of the fibrinogen staining (Fig. 3A, lower panels). The results indicate viable endothelial cells in PBS-injected striatum but fragmented cells in excitotoxin-injected striatum. Representative RECA-1 ir with Cyclo-VEGI application with QUIN or applied alone showed similar RECA-1 staining as for control. These data suggest abnormalities in endothelial cell structure are associated with QUIN-striatal injection with some restoration of structure following Cyclo-VEGI treatment.

### Effects of Cyclo-VEGI on neuronal viability

**Figure 4:**
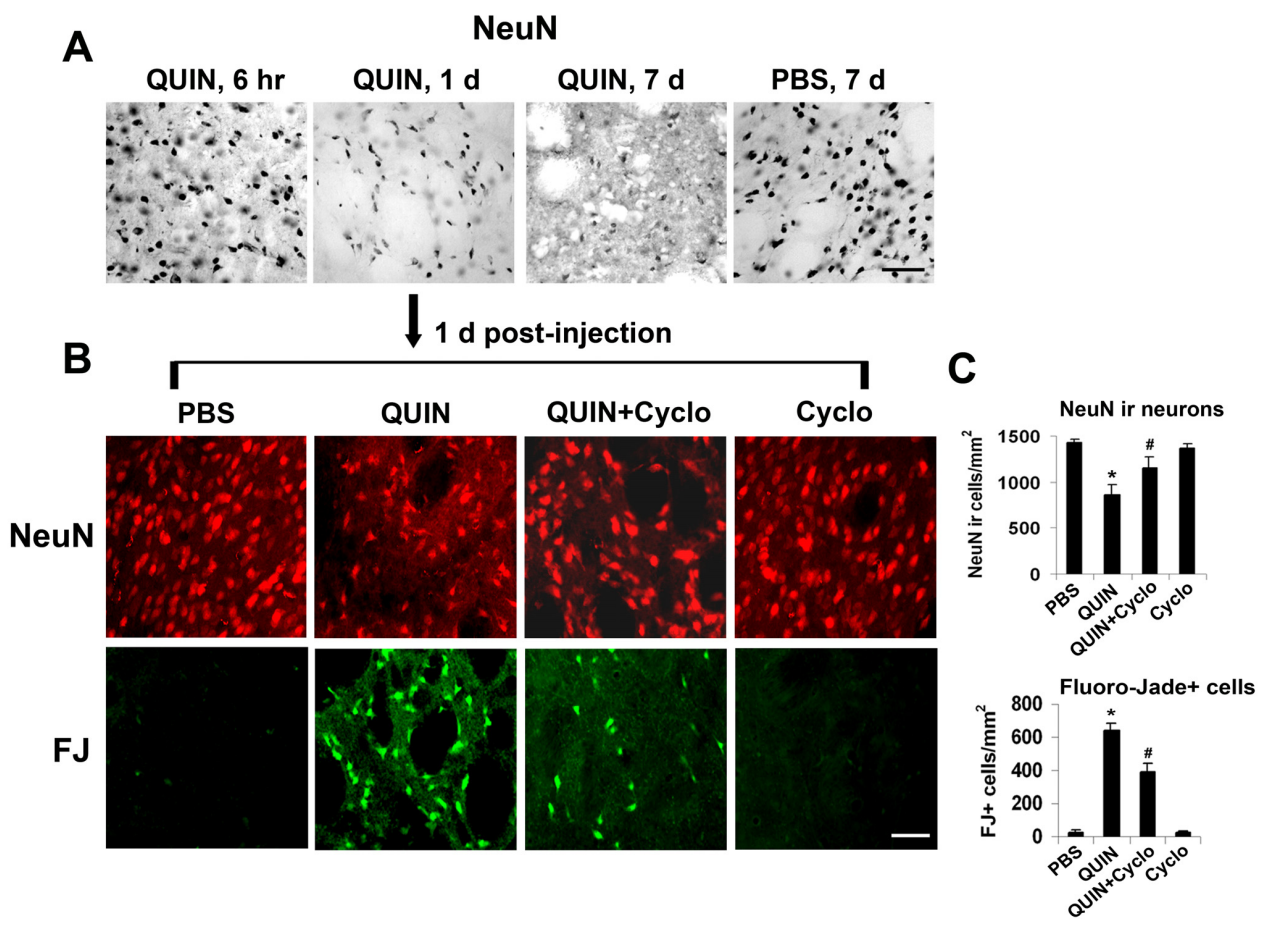
**Effects of Cyclo-VEGI on viability of striatal neurons**. (A) Time-dependent changes in numbers of NeuN (+)ve neurons with QUIN injection (6 hr, 1 and 7 d) and PBS injection at 7 d. Scale bar = 100 μm. (B) Photomicrographs of NeuN (+)ve neurons (upper row) and Fluoro-Jade B (+)ve degenerating neurons (lower row) in striatum from PBS (left panels), QUIN-injected (left middle panels), QUIN-injected rats with Cyclo-VEGI treatment (right middle panels), and Cyclo-VEGI alone (right panels) at 1 day post-injection. Scale bar represents 60 μm. (C) Quantification of the number of NeuN (+)ve neurons (upper bar graph) and Fluoro-Jade B (+)ve degenerating neurons (lower bar graph) in striatum for the four procedures. FJ; Fluoro-Jade B. Data are mean ± SEM (n = 5 animals/group). *p < 0.05 compared with PBS. ^#^*p *< 0.05 compared with QUIN-injected rats.

An important end-point of this study was to examine if antagonism of VEGF receptor expression was neuroprotective in excitotoxin-injected brain. This question was assessed in two ways; measurement of numbers of viable striatal neurons using NeuN as a marker and determination of numbers of damaged neurons using Fluoro-Jade B as a marker. We first examined time-dependence of neuronal viability (NeuN marker) using the same time points as for gliosis (Fig. 1). Representative NeuN staining (Fig. 4A) shows a marked loss of striatal neurons at 1 and 7 d post-QUIN injection compared with the early time point of QUIN injection (6 hr) or after long-term PBS injection (7 d).

Representative NeuN ir shows a considerable attenuation in numbers of neurons following QUIN injection compared with PBS control (Fig. 4B, upper panels). Some recovery in numbers of striatal neurons was evident with Cyclo-VEGI treatment with excitotoxin. Cyclo-VEGI alone showed similar NeuN ir as for PBS control. Quantification of data is presented in Fig. 4C (upper bar graph) with numbers of neurons reduced by 39% (QUIN vs PBS; n = 5 animals/group). Cyclo-VEGI significantly increased numbers of NeuN (+)ve striatal neurons by 33% (QUIN + Cyclo-VEGI vs QUIN). Numbers of neurons with Cyclo-VEGI alone were similar to PBS control.

Representative Fluoro-Jade B staining is shown (Fig. 4B, lower panels). Little or no Fluoro-Jade B (+)ve cells were evident in control or with Cyclo-VEGI administered alone. Marked striatal damage was observed following QUIN injection with Cyclo-VEGI effective in diminishing the marker of neuronal damage when added with excitotoxin. Overall (n = 5 animals/group), Cyclo-VEGI reduced Fluoro-Jade B (+)ve cells by 38% representing a significant effect to protect striatal neurons from damage (Fig. 4C, lower bar graph).

## Discussion

This study is the first report that excitotoxin-induced striatal lesion induces VEGF-mediated signaling as part of an overall inflammatory response. Evidence is provided that pharmacological inhibition of VEGF receptors leads to reduced microgliosis and an increased integrity of BBB and that modulation of these processes link neuronal viability with excitotoxic insult. As discussed below, the results implicate activation of microglial cells as a mechanism for induction of striatal neurodegeneration. Furthermore, VEGF-dependent signaling pathways are identified as candidates for pharmacological modulation to reduce inflammatory reactivity and confer neuroprotection under excitotoxic conditions.

Cyclo-VEGI showed a wide spectrum of effects in QUIN-injected brain including inhibition of multiple processes including VEGF expression, gliosis, impairment and leakiness of BBB and neuronal damage. This compound, like other VEGF receptor antagonists, binds to both VEGF1 (Flt-1) and VEGF2 (Flk-1)-type receptors [23,29] likely reflecting the similar tyrosine kinase structures of the two receptors. The single staining data (Fig. 1B) and double staining results (Fig. 2A) suggests that resident microglia, or macrophage-like immune responding cells, are the primary cells expressing VEGF and which are targeted by Cyclo-VEGI. Furthermore, the present data suggests microglia express VEGFR1 consistent with results from previous studies [17,18]. In this case Cyclo-VEGI binding to VEGFR-1 in activated microglia could inhibit cellular reactivity and VEGF production leading to a reduced vascular permeability for fibrinogen infiltration into striatum. A lower extravasation of fibrinogen would, in a positive feedback manner, attenuate microglial activation and amplification of inflammatory responses. This autocrine scheme would be consistent with the finding of altered RECA-1 staining patterns (insets, Fig. 3) indicating abnormalities in endothelial cells in QUIN-injected striatum with some recovery provided by Cyclo-VEGI treatment. However, protection against endothelial cell damage and BBB leakiness could also be provided from Cyclo-VEGI binding to VEGF receptors on endothelial cells. In this case, paracrine effects of microglial VEGF acting on endothelium would be diminished with effects of the VEGF antagonist directed at vasculature. At present, the relative extents of microglial autocrine and paracrine (vascular actions) in excitotoxin-injected brain are not known.

The present results indicate reactive microglia could serve as a link between excitotoxic insult and striatal neurodegeneration. In this event, damage to striatal neurons could result from activated microglial secretion of an assemblage of inflammatory mediators. The specific nature of mediators was not addressed but likely candidates would include proinflammatory cytokines and reactive oxygen and nitrogen species. It can be speculated that QUIN-injection could have dual effects to both increase microglial secretion of VEGF and expression of VEGFR-1 which would provide both a chemotactic cellular stimulus and also amplify inflammatory responses. However, our results do not preclude QUIN injection causing a direct and rapid excitotoxic striatal injury contributing to overall neuronal damage. Interestingly, such a process could also initiate a microglial chemotactic response mediated by factors such as ATP released from damaged neurons [30,31]. Thus, Cyclo-VEGI could still be effective in inhibiting microglial mobility and chemotaxis with direct excitotoxicity induced by striatal injection of QUIN. An interesting possibility for future study would be to investigate inflammatory responses to excitotoxic insult *in vivo *using a soluble protein treatment to sequester VEGF [32,33].

It is important to note that the findings of deleterious VEGF effects in QUIN-injected intact animals are not demonstrated *in vitro *where VEGF is reported to enhance viability of cultured neurons against excitotoxic insult [34,35]. Neurons can express both VEGFR-1 and VEGFR-2 with evidence suggesting that VEGF binding to the latter receptor confers neuroprotection [34]. The underlying reasons for the differential VEGF effects are not known, however, one possibility is that in the absence of glial stimulation, binding of VEGF to neurons is neurotrophic. Our findings in intact animals suggest the utility of future *in vitro *experiments to examine effects of VEGF applied in co-cultures of neurons and glia which are exposed to excitotoxic conditions.

## Conclusion

Excitotoxicity is manifest under conditions of elevated glutamate and thus could contribute to a spectrum of pathological conditions including ones in neurodegenerative diseases. However, VEGF may have multifactorial functional roles in different diseases such as demonstrated with beneficial effects of VEGF treatment in amyotrophic lateral sclerosis [16] and detrimental effects in multiple sclerosis [36] or where excitotoxic conditions prevail. Overall, the present results are consistent with an acute excitotoxic insult inducing extensive inflammatory reactivity which includes vascular leakiness and diminished neuronal viability. The findings from this work suggest that in addition to using antagonists for glutamate sub-type receptors, amelioration of brain excitotoxicity could be derived from inhibition of VEGFR-1-dependent microglial inflammatory signaling pathways.

## Competing interests

The authors declare that they have no competing interests.

## Authors' contributions

JKR designed research, performed research, analyzed data, and wrote the manuscript.

JGM designed research, analyzed data, and wrote the manuscript.

All authors read and approved the final manuscript.

## References

[B1] Greenamyre JT, Young AB (1989). Excitatory amino acids and Alzheimer's disease. Neurobiol Aging.

[B2] Beal MF, Ferrante RJ, Swartz KJ, Kowall NW (1991). Chronic quinolinic acid lesions in rats closely resemble Huntington's disease. J Neurosci.

[B3] Bal-Price A, Brown GC (2001). Inflammatory neurodegeneration mediated by nitric oxide from activated glia-inhibiting neuronal respiration, causing glutamate release and excitotoxicity. J Neurosci.

[B4] Lehrmann E, Molinari A, Speciale C, Schwarcz R (2001). Immunohistochemical visualization of newly formed quinolinate in the normal and excitotoxically lesioned rat striatum. Exp Brain Res.

[B5] Siao CJ, Tsirka SE (2002). Tissue plasminogen activator mediates microglial activation via its finger domain through annexin II. J Neurosci.

[B6] Ryu JK, Kim SU, McLarnon JG (2004). Blockade of quinolinic acid-induced neurotoxicity by pyruvate is associated with inhibition of glial activation in a model of Huntington's disease. Exp Neurol.

[B7] Ryu JK, Choi HB, McLarnon JG (2005). Peripheral benzodiazepine receptor ligand PK11195 reduces microglial activation and neuronal death in quinolinic acid-injected rat striatum. Neurobiol Dis.

[B8] Tikka T, Fiebich BL, Goldsteins G, Keinanen R, Koistinaho J (2001). Minocycline, a tetracycline derivative, is neuroprotective against excitotoxicity by inhibiting activation and proliferation of microglia. J Neurosci.

[B9] Santamaría A, Salvatierra-Sánchez R, Vázquez-Román B, Santiago-López D, Villeda-Hernández J, Galván-Arzate S, Jiménez-Capdeville ME, Ali SF (2003). Protective effects of the antioxidant selenium on quinolinic acid-induced neurotoxicity in rats: in vitro and in vivo studies. J Neurochem.

[B10] Ryu JK, Choi HB, McLarnon JG (2006). Combined minocycline plus pyruvate treatment enhances effects of each agent to inhibit inflammation, oxidative damage and neuronal loss in an excitotoxic animal model of Huntington's disease. Neuroscience.

[B11] Lipton SA, Gu Z, Nakamura T (2007). Inflammatory mediators leading to protein misfolding and uncompetitive/fast off-rate drug therapy for neurodegenerative disorders. Int Rev Neurobiol.

[B12] Pearson VL, Rothwell NJ, Toulmond S (1999). Excitotoxic brain damage in the rat induces interleukin-1beta protein in microglia and astrocytes: correlation with the progression of cell death. Glia.

[B13] Leonoudakis D, Braithwaite SP, Beattie MS, Beattie EC (2004). TNFalpha-induced AMPA-receptor trafficking in CNS neurons; relevance to excitotoxicity?. Neuron Glia Biol.

[B14] Gary DS, Bruce-Keller AJ, Kindy MS, Mattson MP (1998). Ischemic and excitotoxic brain injury is enhanced in mice lacking the p55 tumor necrosis factor receptor. J Cereb Blood Flow Metab.

[B15] Ferrara N (2001). Role of vascular endothelial growth factor in regulation of physiological angiogenesis. Am J Physiol Cell Physiol.

[B16] Storkebaum E, Lambrechts D, Carmeliet P (2004). VEGF: once regarded as a specific angiogenic factor, now implicated in neuroprotection. Bioessays.

[B17] Barleon B, Sozzani S, Zhou D, Weich HA, Mantovani A, Marmé D (1996). Migration of human monocytes in response to vascular endothelial growth factor (VEGF) is mediated via the VEGF receptor flt-1. Blood.

[B18] Forstreuter F, Lucius R, Mentlein R (2002). Vascular endothelial growth factor induces chemotaxis and proliferation of microglial cells. J Neuroimmunol.

[B19] Croll SD, Ransohoff RM, Cai N, Zhang Q, Martin FJ, Wei T, Kasselman LJ, Kintner J, Murphy AJ, Yancopoulos GD, Wiegand SJ (2004). VEGF-mediated inflammation precedes angiogenesis in adult brain. Exp Neurol.

[B20] Storkebaum E, Carmeliet P (2004). VEGF: a critical player in neurodegeneration. J Clin Invest.

[B21] Weis SM, Cheresh DA (2005). Pathophysiological consequences of VEGF-induced vascular permeability. Nature.

[B22] Paxinos G, Watson C (1986). The rat brain in stereotaxic coordinates.

[B23] Bikfalvi A (2004). Recent developments in the inhibition of angiogenesis: examples from studies on platelet factor-4 and the VEGF/VEGFR system. Biochem Pharmacol.

[B24] Zilberberg L, Shinkaruk S, Lequin O, Rousseau B, Hagedorn M, Costa F, Caronzolo D, Balke M, Canron X, Convert O, Laïn G, Gionnet K, Goncalvès M, Bayle M, Bello L, Chassaing G, Deleris G, Bikfalvi A (2003). Structure and inhibitory effects on angiogenesis and tumor development of a new vascular endothelial growth inhibitor. J Biol Chem.

[B25] Weis S, Cui J, Barnes L, Cheresh D (2004). Endothelial barrier disruption by VEGF-mediated Src activity potentiates tumor cell extravasation and metastasis. J Cell Biol.

[B26] Ryu JK, Tran KC, McLarnon JG (2007). Depletion of neutrophils reduces neuronal degeneration and inflammatory responses induced by quinolinic acid in vivo. Glia.

[B27] Dickstein DL, Biron KE, Ujiie M, Pfeifer CG, Jeffries AR, Jefferies WA (2006). Abeta peptide immunization restores blood-brain barrier integrity in Alzheimer disease. FASEB J.

[B28] Schmued LC, Hopkins KJ (2000). Fluoro-Jade B: a high affinity fluorescent marker for the localization of neuronal degeneration. Brain Res.

[B29] Koyama J, Miyake S, Sasayama T, Kondoh T, Kohmura E (2007). Effect of VEGF Receptor Antagonist (VGA1155) on Brain Edema in the Rat Cold Injury Model. Kobe J Med Sci.

[B30] Inoue K (2002). Microglial activation by purines and pyrimidines. Glia.

[B31] McLarnon JG (2005). Purinergic mediated changes in Ca2+ mobilization and functional responses in microglia: effects of low levels of ATP. J Neurosci Res.

[B32] Aiello LP, Pierce EA, Foley ED, Takagi H, Chen H, Riddle L, Ferrara N, King GL, Smith LE (1995). Suppression of retinal neovascularization in vivo by inhibition of vascular endothelial growth factor (VEGF) using soluble VEGF-receptor chimeric proteins. Proc Natl Acad Sci USA.

[B33] van Bruggen N, Thibodeaux H, Palmer JT, Lee WP, Fu L, Cairns B, Tumas D, Gerlai R, Williams SP, Campagne M van Lookeren, Ferrara N (1999). VEGF antagonism reduces edema formation and tissue damage after ischemia/reperfusion injury in the mouse brain. J Clin Invest.

[B34] Matsuzaki H, Tamatani M, Yamaguchi A, Namikawa K, Kiyama H, Vitek MP, Mitsuda N, Tohyama M (2001). Vascular endothelial growth factor rescues hippocampal neurons from glutamate-induced toxicity: signal transduction cascades. FASEB J.

[B35] Svensson B, Peters M, König HG, Poppe M, Levkau B, Rothermundt M, Arolt V, Kögel D, Prehn JH (2002). Vascular endothelial growth factor protects cultured rat hippocampal neurons against hypoxic injury via an antiexcitotoxic, caspase-independent mechanism. J Cereb Blood Flow Metab.

[B36] Proescholdt MA, Jacobson S, Tresser N, Oldfield EH, Merrill MJ (2002). Vascular endothelial growth factor is expressed in multiple sclerosis plaques and can induce inflammatory lesions in experimental allergic encephalomyelitis rats. J Neuropathol Exp Neurol.

